# Cesarean delivery in Iran: a population-based analysis using the Robson classification system

**DOI:** 10.1186/s12884-022-04517-1

**Published:** 2022-03-08

**Authors:** Maryam Pourshirazi, Mohammad Heidarzadeh, Mahshid Taheri, Habibollah Esmaily, Farah Babaey, Nasrin Talkhi, Leila Gholizadeh

**Affiliations:** 1 Department of Hospital Management and Clinical Services Excellence, Mashhad University of Medical Sciences, Mashhad, Iran; 2 Neonatal Health Office, Ministry of Health and Medical Education, Tehran, Iran; 3 Department of Neonatology, Tabriz University of Medical Sciences, Tabriz, Iran; 4 Department of Hospital Management and Clinical Services Excellence, Ministry of Health and Medical Education, Tehran, Iran; 5 Social Determinants of Health Research Center, Mashhad University of Medical Sciences, Mashhad, Iran; 6 Department of biostatistics, School of Health, Mashhad university of Medical Sciences, Mashhad, Iran; 7 Faculty of Health, University of Technology, Sydney, NSW Australia

**Keywords:** Cesarean section, Rate, Robson classification, Ten-group, Audit, Iran

## Abstract

**Background:**

The rise of Cesarean Sections (CS) is a global concern. In Iran, the rate of CS increased from 40.7% in 2005 to 53% in 2014. This figure is even higher in the private sector.

**Objective:**

To analyze the CS rates in the last 2 years using the Robson Classification System in Iran**.**

**Methods:**

A retrospective analysis of all in-hospital electronically recorded deliveries in Iran was conducted using the Robson classification. Comparisons were made in terms of the type of hospital, CS rate, and obstetric population, and contributions of each group to the overall cesarean deliveries were reported.

**Results:**

Two million three hundred twenty-two thousand five hundred women gave birth, 53.6% delivered through CS. Robson group 5 was the largest contributing group to the overall number of cesarean deliveries (47.1%) at a CS rate of 98.4%. Group 2 and 1 ranked the second and third largest contributing groups to overall CSs (20.6 and 10.8%, respectively). The latter groups had CS rates much higher than the WHO recommendation of 67.2 and 33.1%, respectively. “Fetal Distress” and “Undefined Indications” were the most common reasons for cesarean deliveries at CS rates of 13.6 and 13.4%, respectively. There was a significant variation in CS rate among the three types of hospitals for Robson groups 1, 2, 3, 4, and 10.

**Conclusion:**

The study revealed significant variations in CS rate by hospital peer-group, especially for the private maternity units, suggesting the need for further attention and audit of the Robson groups that significantly influence the overall CS rate. The study results will help policymakers identify effective strategies to reduce the CS rate in Iran, providing appropriate benchmarking to compare obstetric care with other countries that have better maternal and perinatal outcomes.

**Supplementary Information:**

The online version contains supplementary material available at 10.1186/s12884-022-04517-1.

## Introduction

Increased prevalence of cesarean section (CS) is a global concern during recent decades and reducing CS rates (Natural Childbirth Promotion) is one of the priorities of Iran’s Health Reform Plan [1–3]. World health organization (WHO) stated that the CS rate higher than 10% nationwide does not improve maternal and neonatal outcomes [4]. Medically indicated CS will save the mother and newborn, but unnecessary CS is associated with higher morbidity [5] and healthcare costs [6]. Iran’s CS rates have dramatically increased since the beginning of the current century. Reports of the Iran's Ministry of Health demonstrate that CS rates were 40.7% in 2005, reaching 53% in 2014 [7, 8]. This figure is even higher in the private sector, with the rate as high as 90% [7]. Since 2014, the promotion of natural childbirth has been a core aim of the healthcare reform in Iran. Thus, ongoing national programs have led to a decrease in the rate of unnecessary CS, although the targeted values are not achieved [9]. Analysis of CS rates in Iran using an appropriate classification tool can be an important step in reducing unnecessary CS rates [4].

To classify and compare CS rates, the Robson Ten-Group Classification System (Table 1) has been used since 2001 in many countries. The system is based on five simple obstetric parameters, including parity, number of foetuses, previous caesarean section, onset of labour, gestational age, and fetal presentation, and is considered easy to implement [10]. Within each of these ten groups, CS indications can be compared. Robson Classification, the most appropriate tool to compare CS rates across health care settings, allows policy-makers to analyze the overall status of obstetric care and birth interventions at the hospital, regional, and national levels [11].

**Table 1:** The Robson Classification

Group	Obstetric Population
1	Nulliparous women with a single cephalic pregnancy, ≥37 weeks gestation in spontaneous labour
2	Nulliparous women with a single cephalic pregnancy, ≥37 weeks gestation who had labour induced or were delivered by CS before labour
2a	Labour induced
2b	Pre-labour CS
3	Multiparous women without a previous CS, with a single cephalic pregnancy, ≥37 weeks gestation in spontaneous labour
4	Multiparous women without a previous CS, with a single cephalic pregnancy, ≥37 weeks gestation who had labour induced or were delivered by CS before labour
4a	Labour induced
4b	Pre-labour CS
5	All multiparous women with at least one previous CS, with a single cephalic pregnancy, ≥37 weeks gestation
6	All nulliparous women with a single breech pregnancy
7	All multiparous women with a single breech pregnancy including women with previous CS(s)
8	All women with multiple pregnancies including women with previous CS(s)
9	All women with a single pregnancy with a transverse or oblique lie, including women with previous CS(s)
10	All women with a single cephalic pregnancy < 37 weeks gestation, including women with previous CS(s)

World Health Organization. Robson Classification: Implementation Manual. 2015

This study reports the prevalence of CS in Iran. The objective is to analyze the CS rates in the last 2 years using the Ten-Group Robson Classification System in Iran; comparisons have been made considering the type of hospitals (public VS private VS other types).

## Materials and methods

### Study setting and design

We conducted a retrospective analysis of all in-hospital deliveries in Iran. Data were obtained from the Iranian Maternal and Neonatal (IMaN) database. IMaN is an established national birth registry database for maternal and neonatal information, administered by the Iranian Ministry of Health. It is the most comprehensive source of information for monitoring maternal and neonatal health, recording all birth-related data electronically in and out of hospitals throughout the country since March 21, 2013 [12]. The collected information includes demographic data, maternal characteristics, medical history, obstetric interventions, pregnancy and delivery complications, and perinatal outcomes. According to the hospital accreditation program implemented in Iran, the validity and reliability of the IMaN registry database are evaluated by randomly checking medical records on a periodic basis. In the past 2 years, the rate of data registration was approximately more than 95%. Additionally, IMaN provides a unique interface for interactive presentation and analysis of the collected information on a Qlikview platform representing data at hospital, medical university and national levels. All the obstetric parameters for applying the Robson Classification, based on the WHO implementation Manual, are available on the IMaN registry database [13].

### Data collection

In the present study, we included all women who had given birth between March 21, 2019, and March 21, 2021, two consecutive years (March 21 marks the beginning of a new year in Iran). Hospitals were assigned to three peer groups of Public, Private, and Other Hospitals. Public hospitals were defined as those in which the government and health insurance cover all medical bills; they are administered by public universities and provide clinical education to students across allied health professions [14]. The midwifery model of care under the supervision of obstetrician is dominant in public hospitals. In most cases, the scope of a midwife’s activities is decided by an obstetrician with multifactorial barriers to midwifery autonomy [15, 16]. In Private hospitals, all delivery costs are self-funded or covered by private health insurance [14] and the maternity care is provided on the basis of biomedical model. During and after labour, an obstetrician should be present and midwives provide care [16]. The obstetrician most often manages deliveries. The category “Other Hospitals” includes settings that are a subset of a specific organization (i.e. charity, affiliated with Islamic Azad University, affiliated with Social Security Organization, and affiliated with Armed Forces) [14]. The structure and the model of maternity care in these hospitals are almost similar to the public settings.

Deliveries were categorized using the Ten-Group Robson Classification System. We described maternal and neonatal characteristics, obstetric interventions, medical conditions, and obstetric outcomes. Then, for each Robson group, we reported the total number of CS, the total number of women delivered, group size to the overall population, CS rate, absolute group contribution to overall CS rate, and relative contribution of the group to overall CS rate for the national level. Rates were calculated and extracted from the IMaN registry database according to the WHO implementation manual.

### Statistical analysis

Data analysis was performed with the statistical software SPSS version 26.0 (SPSS, Chicago IL, USA). Categorical data are shown as frequencies (percentages). One-way ANOVA was used to compare between hospital peer-group for each Robson group. To compare CS indications and report Odds Ratio by hospital peer-group, we applied the Logistic Regression model. The significance level was set at α = 0.05. All methods were carried out in accordance with relevant guidelines and regulations in the declaration - Ethics approval and consent to participate section.

## Results

The total number of women who had given birth in 962 Iranian hospitals affiliated with 63 medical universities was 2,322,500 between 21 March 2019 and 21 March 2021. Characteristics of the obstetric population, medical and obstetric conditions are shown in Table 2. Our study showed that out of 2,322,500 deliveries, 56.8% were delivered in public hospitals, 20.2% in private hospitals, and 23% in other hospitals. Iranian hospitals have a 34.4% rate of low-literate mothers giving birth. Moreover, about 50% of women in the public sector are low-literate. The total number of deliveries stimulated by oxytocin accounted for 10.8%, and only 1.3% involved operative vaginal delivery.

**Table 2:** Characteristics of the obstetric population, medical and obstetric conditions by hospital peer-group in Iran, 21 March 2019–21 March 2021

Characteristics		Public	Private	Other Hospitals	Total Hospitals
		n (%) ^**a**^	n (%) ^**a**^	n (%) ^**a**^	n (%) ^**a**^
**Age (years)**	**< 18**	60,230 (4.6)	5224 (1.1)	13,302 (2.5)	78,756 (3.4)
	**18–35**	993,561 (75.3)	346,966 (73.9)	410,461 (76.9)	1,750,988 (75.4)
	**> 35**	265,347 (20.2)	117,218 (25)	110,191 (20.6)	492,756 (21.2)
**Education Level**	**Low literacy**	649,544 (49.2)	30,568 (6.5)	119,523 (22.4)	799,635 (34.4)
	**High School and Associate Degree**	533,233 (40.4)	194,194 (41.4)	291,971 (54.7)	1,019,398 (43.9)
	**Bachelor and Above**	140,624 (10.7)	249,907 (53.2)	123,917 (23.2)	514,448 (22.2)
	**Unknown**	17,487 (1.3)	3370 (0.7)	5578 (1)	26,435 (1.1)
**Nationality**	**Iranian**	1,230,293 (93.3)	462,596 (98.6)	502,877 (94.2)	2,195,766 (94.5)
	**Immigrant**	88,845 (6.7)	6812 (1.4)	31,077 (5.8)	126,734 (5.5)
**Obstetric and Medical Conditions**	**Preeclampsia/ Eclampsia**	35,419 (2.7)	7986 (2.7)	8311 (1.6)	51,716 (2.2)
	**Gestational Diabetes**	59,897 (4.5)	29,748 (6.3)	25,914 (4.9)	115,559 (5)
	**Thyroid Disorders**	96,336 (7.3)	77,749 (16.6)	57,665 (10.8)	231,750 (10)
	**Hypertension**	22,027 (1.7)	6931 (1.5)	6489 (1.2)	35,447 (1.5)
	**Heart Disease**	8385 (0.6)	4552 (1)	4257 (0.8)	17,194 (0.7)
**Obstetric Interventions**	**Oxytocin**	158,698 (12)	30,920 (6.6)	62,239 (11.7)	251,857 (10.8)
	**Vaginal Operative Delivery** ^b^	7608 (1)	2569 (2.6)	4905 (1.9)	15,082 (1.3)
	**Episiotomy** ^b^	268,953 (35.2)	49,683 (49.4)	89,100 (34.4)	407,736 (36.3)
	**Medical Pain Relief for Labour**	52,590 (4)	18,083 (3.9)	38,101 (7.1)	108,774 (4.7)
	**Non-medical Pain Relief for Labour**	199,665 (15.1)	28,462 (6)	86,795 (16.3)	314,922 (13.6)
**Obstetric complications**	**Blood Transfusion**	4896 (0.4)	742 (0.2)	1010 (0.2)	6648 (0.3)
	**3rd to 4th degree Perineal Tear** ^b^	1382 (0.2)	141 (0.1)	292 (0.1)	1815 (0.2)
**Gestational Age (weeks)** ^c^	**< 32**	31,279 (2.3)	3791 (0.8)	5083 (0.9)	40,153 (1.7)
	**32–36**	126,055 (9.4)	29,388 (6.2)	29,656 (5.5)	185,099 (7.8)
	**≥37**	1,183,554 (88.3)	444,860 (93)	506,250 (93.6)	2,134,664 (90.5)
**Birth weight (g)** ^c^	**< 500**	3105 (0.2)	630 (0.1)	882 (0.2)	4617 (0.2)
	**500–749**	7294 (0.5)	1075 (0.2)	1323 (0.2)	9692 (0.4)
	**750–999**	5415 (0.4)	598 (0.1)	831 (0.2)	6844 (0.3)
	**1000–1499**	13,657 (1)	1489 (0.3)	1909 (0.4)	17,055 (0.7)
	**1500–2499**	105,848 (7.9)	25,499 (5.3)	28,533 (5.3)	159,880 (6.8)
	**2500–3999**	1,187,663 (88.6)	442,889 (92.7)	501,279 (92.7)	2,131,831 (90.4)
	**≥4000**	17,586 (1.3)	5788 (1.2)	6144 (1.1)	29,518 (1.3)
**Perinatal Outcome** ^c^	**Apgar < 7 at 5 min**	26,839 (2)	3635 (0.8)	4951 (0.9)	35,425 (1.5)
	**Stillbirth**	13,944 (1)	2206 (0.5)	2983 (0.6)	19,133 (0.8)
	**Neonatal Death (2 h postpartum)**	3584 (0.3)	468 (0.1)	609 (0.1)	4661 (0.2)
	**Admitted to Neonatal Unit**	140,136 (10.5)	19,612 (4.1)	30,186 (5.6)	189,934 (8)
**Deliveries**		1,319,138 (56.8)	469,408 (20.2)	533,954 (23)	2,322,500 (100)
**Births**		1,340,888 (56.8)	478,039 (20.2)	540,989 (23)	2,359,916 (100)

^a^ The sum of the percentages may not be 100 due to errors introduced by rounding

^b^ Vaginal deliveries are considered as the denominator for calculating the percentages

^c^ Births are considered as the denominator for calculating the percentages

The total rate of Cesarean Sections was 51.6%. All the deliveries were categorized into the 10 Robson groups (Table 3). There was no missing data on Robson Classification, as the record of the Robson criteria is obligatory for birth registries on the IMaN.

**Table 3:** Cesarean Sections by the Robson classification system in Iran, 21 March 2019–21 March 2021

Group	Number of CS in group	Number of women in group	Obstetric population ^a^ (%)	CS rate ^b^ (%)	Absolute contribution ^c^ (%)	Relative contribution ^d^ (%)
1	129,245	390,791	16.8	33.1	5.6	10.8
2	246,619	366,822	15.8	67.2	10.6	20.6
2a	56,913	172,737	7.4	33	2.5	4.8
2b	189,706	194,085	8.4	97.7	8.2	15.8
3	34,807	553,021	23.8	6.3	1.5	2.9
4	40,504	174,464	7.5	23.2	1.7	3.4
4a	11,778	140,322	6	8.4	0.5	1
4b	28,726	34,142	1.5	84.1	1.2	2.4
5	564,782	574,218	24.7	98.4	24.3	47.1
6	35,618	37,419	1.6	95.2	1.5	3
7	21,549	24,347	1.1	88.5	0.9	1.8
8	30,974	35,175	1.5	88	1.3	2.6
9	4414	4574	0.2	100	0.2	0.4
10	90,579	161,669	7	56	3.9	7.5
Total	1,199,091	2,322,500	100 ^e^	51.6	51.6	100 ^e^

^a^ Obstetric Population (%) = n of women in the group / total N women delivered in the hospital ×100

^b^ CS rate (%) = n of CS in the group / total N of women in the group ×100

^c^ Absolute contribution (%) = n of CS in the group / total N of women delivered in the country × 100

^d^ Relative contribution (%) = n of CS in the group / total N of CS in the country × 100

^e^ The sum of the percentages may not be 100 due to errors introduced by rounding

The largest contributing group to the total number of cesarean sections was Robson group 5, which consisted of all multiparous women with at least one previous CS, with a single cephalic pregnancy and ≥ 37 weeks gestation. The size of this group accounted for one-fourth of the total obstetric population (24.7%), identified as the largest group in the whole study population. Its CS rate was 98.4%, with the largest absolute contribution (564,782 CS of 2,322,500, 24.3%).

The second-largest contribution group to the overall number of cesarean deliveries was Robson group 2, consisting of nulliparous women with a single cephalic pregnancy, and ≥ 37 weeks gestation who were induced or delivered by CS before labour. The obstetric population of this group accounted for 15.8% of the total population, with a CS rate of 67.2%. Group 2 contributed to the 20.6% of all cesarean deliveries, of which 15.8% belonged to group 2b.

Nulliparous women with a single cephalic pregnancy and ≥ 37 weeks gestation who had spontaneous labour (Robson group 1) were ranked the third-largest contribution to the overall CSs (10.8%). This group accounted for 16.8% of the total obstetric population, with a CS rate of 33.1%. It should be noted that pre-labour CS rates at 38 to 39 weeks gestation accounted for about 8.8% of the total deliveries.

Analyses by hospital peer-group are shown in Table 4. There was a significant statistical difference (*p* <  0.05) in CS rates between private hospitals and the other two peer groups in all Robson groups (excluding group 9). The partial eta squared results by one-way ANOVA indicated that the effect sizes for CS rates in Robson groups 2, 4, 10, 1, 8, 3, 5, 6, and 7, respectively, were larger between hospital peer groups, which is demonstrative of group mean differences.

**Table 4:** Comparison of the CS rates of each group of the Robson classification by hospital peer-group in Iran, 21 March 2019–21 March 2021

Group	Public (*n* = 602)	Private (*n* = 172)	Other Hospitals (*n* = 188)	Partial Eta Squared	*P* value
	Mean ± SD	Mean ± SD	Mean ± SD		
1	28.9 ± 9.7 ^a^	50.7 ± 18.4 ^b^	26.8 ± 11.6 ^a^	0.4	< 0.001
2	46.7 ± 13.2 ^a^	83.1 ± 14.6 ^b^	53.5 ± 22 ^a^	0.4	< 0.001
3	6.4 ± 2.6 ^a^	12.7 ± 8.9 ^b^	4.8 ± 3.1 ^a^	0.3	< 0.001
4	19.5 ± 9 ^a^	48.2 ± 19.5 ^b^	26.8 ± 11.6 ^a^	0.4	< 0.001
5	97.7 ± 2.5 ^a^	99.5 ± 0.3 ^b^	98.7 ± 1.4 ^b^	0.1	< 0.001
6	96.3 ± 3.6 ^a^	98.8 ± 0.9 ^b^	97.3 ± 2.6	0.1	< 0.001
7	91.8 ± 4.2 ^a^	95.2 ± 3.7 ^b^	93.5 ± 4.4	0.1	0.001
8	87 ± 6.6 ^a^	96.6 ± 2.4 ^b^	89.4 ± 7.1 ^a^	0.3	< 0.001
9	100 ^a^	100 ^a^	100 ^a^	0	1
10	51 ± 9.8 ^a^	70.3 ± 11.6 ^b^	49.6 ± 11 ^a^	0.4	< 0.001

^a,b^ Non-identical letters are significantly different (*P* value < 0.05)

Table 5 represents CS indications by hospital peer-group for two consecutive years in Iran. “Fetal Distress” and “Undefined Indications” were the most common reasons for cesarean deliveries at CS rates of 13.6 and 13.4%, respectively. The highest prevalence of Fetal Distress was found in public hospitals. Private hospitals were 2.5 times more likely to have Undefined Indications compared to other hospitals. However, public hospitals were 49% less likely than private hospitals to have Undefined Indications.

**Table 5:** Comparison of the CS indications by hospital peer-group in Iran, 21 March 2019–21 March 2021

Indication	Public	Private	Other Hospitals	OR (CI, 95%) Public VS Other Hospitals	OR (CI, 95%) Private VS Other Hospitals	OR (CI, 95%) Public VS Private
	n (%)	n (%)	n (%)			
**Repeated CS**	308,701 (55.6)	162,615 (44.1)	163,522 (59.5)	0.85 (0.84, 0.86)	0.53 (0.53, 0.54)	1.58 (1.57, 1.60)
**Fetal Distress**	95,218 (17.1)	34,713 (9.4)	32,935 (12)	1.52 (1.50, 1.54)	0.76 (0.75, 0.77)	1.99 (1.96, 2.01)
**Dystocia**	22,063 (4)	14,869 (4)	12,555 (4.6)	0.86 (0.84, 0.88)	0.87 (0.85, 0.89)	0.98 (0.96, 1.00)
**Placenta and Cord Conditions**	11,703 (2.1)	7311 (2)	3540 (1.3)	1.65 (1.58, 1.71)	1.55 (1.48, 1.61)	1.06 (1.03, 1.09)
**Multiple Pregnancy**	12,430 (2.2)	5750 (1.6)	3963 (1.4)	1.75 (1.68, 1.82)	1.21 (1.16, 1.26)	1.44 (1.40, 1.49)
**Malpresentation**	38,353 (6.9)	16,425 (4.5)	14,078 (5.1)	1.37 (1.34, 1.40)	0.86 (0.84, 0.88)	1.59 (1.56, 1.62)
**Maternal Request**	4153 (0.7)	53,494 (14.5)	19,847 (7.2)	0.097 (0.094, 0.100)	2.18 (2.14, 2.21)	0.044 (0.043, 0.046)
**Undefined Indications**	62,692 (11.3)	73,658 (20)	24,503 (8.9)	1.30 (1.28, 1.32)	2.55 (2.51, 2.59)	0.51 (0.50, 0.51)
***P*** **value**	< 0.001					

Each indication was compared with the rest of the indications to calculate Odds Ratio (OR)

Tables S1 and S2 show mean CS rates in groups 1 and 2 for 63 Iranian medical universities and the number of hospitals affiliated with each university by hospital peer-group (See Additional files 1 and 2).

## Discussion

The current study analyzed 2,322,500 in-hospital deliveries in Iran and categorized them using the Robson classification system to describe and compare cesarean section rates. Between 21 March 2019 and 21 March 2021, the CS rates was 51.6% in Iran, which is considerably higher than many countries as well as the WHO recommended rates. The rates of cesarean sections in nulliparous women in Iran in two consecutive years was 52.4%. Although Robson group 5 was the largest contributing group to the total CSs (47.1%), the government’s approach is to reduce the CS rates in nulliparous women based on Natural Childbirth Promotion program [17, 18]. Given that 31.4% of all cesarean deliveries were belonged to groups 1 and 2, the application of the Robson classification system provides a better understanding of the CS rates and population variations among maternity units across provincial and national levels.

Our study showed that Robson groups 1 to 4 accounted for about 63.9% of the obstetric population in Iran. Such that 37.6% of the population was attributed to nulliparous women with a singleton, term, cephalic pregnancy, and no previous cesarean delivery (Robson Groups 1 and 2). The Netherlands and the Nordic countries such as Iceland, and Norway are reported to have low CS rates across all groups of Robson with high quality of obstetric care resulting in good perinatal outcomes. In the Netherlands, for instance, 39.9% of the total deliveries contribute to Robson group 1 and 2, which is similar to the population of the same groups in Iran. However, the CS rates in these groups vary significantly between the two countries. In other words, CS rates in groups 1 and 2 in the Netherlands are 9.6, and 28.9% (within the WHO recommended ranges) [19, 20], while the corresponding proportions are 33.1 and 67.2%, respectively, in Iran.

In recent years, many attempts have been made to develop and implement interventions to reduce unnecessary CS in Iran, including setting up mother-friendly hospitals, creating standard labour and birth protocols, and providing childbirth preparation classes for women, midwives, and obstetricians through Natural Childbirth Promotion. However, despite this program being effective, the rate remains high [21]. According to our findings, the private sector significantly influenced the rate of CS in groups 1 and 2. This may be because most private hospitals practice a biomedical model of care, and financial incentives motivate obstetricians to perform CS without clinical indications, therefore ignoring protocol implementation. Moreover, pain-related fears of labour and vaginal birth among women and their financial capability to access the desired healthcare and services lead them to favor cesarean section. Consequently, planned cesareans accounted for a significant proportion of surgical deliveries among nulliparous women (group 2b) in our study.

Legal issues related to birth injuries may contribute to the low rates of operative vaginal delivery and non-standard rates of induction of labour in the country. Currently, only obstetricians are authorized to perform operative vaginal deliveries, and only a few of the new generation are skilled enough to do this procedure. Therefore, most obstetricians prefer emergency or planned CS to trial of labour. Furthermore, low literacy in the public sector in Iran is representative of the low tendency of women for vaginal birth. Choosing the mode of delivery appears to be influenced by health literacy. A study in Bangladesh showed that health awareness, health education, and better health communication via mass media foster mothers’ ability to access appropriate health care advice on options for a safe birth and other related issues [22].

In a Canadian study using the Robson classification, group 5 was the largest contributing group to the overall cesarean deliveries (36.6%), and groups 2 and 1 ranked as the second-and third-largest contribution (15.7 and 14.1%, respectively), the same pattern was identified in our study [23]. In a study conducted in the Netherlands, about 71% of women in group 5 experienced a trial of labour, of which only 43% required cesarean delivery. Netherlands is known as a country with low CS rates and good maternal and perinatal outcomes [20]. The organization of maternity care in the Netherlands is based on the model of midwifery-led continuity of care [24]. However, despite the large obstetric population of group 5 in Iran, Vaginal Birth After Cesarean (VBAC) is not a priority for Iran’s government in its healthcare reform policy. It is mostly due to a lack of skills and a fear of legal repercussions. In addition, VBAC is specifically permitted in referral hospitals, but because of the high load of patients in these settings, the priority of the hospital does not lie in underperforming VBAC.

Given that in Iran, the majority of the obstetric population attributes to Robson groups 1 and 2, reducing CSs among nulliparous women would be highly effective in reducing the total national CS rate, as these women will contribute to group 5 population in the future. This could be achieved through quality improvement strategies focused on interventions such as auditing the hospitals by the use of the Robson classification system, review of patient records for CS indications, feedback for the health providers, revision and monitoring of labour and delivery management guidelines, and application of benchmarking and best practices. Iceland implemented a CS auditing program using the Robson classification in 2000 which had led to a significant decrease in CS rates by the end of 2011 [19]. This is an evidence-based example of achieving positive results by benchmarking and implementing a monitoring program to reduce cesarean deliveries.

The results of the present study show that the private sector plays an important role in the rise of CS rates in Robson groups 1, 2, 3, 4, and 10. A typical CS delivery scenario in the Iranian private sector involves planned nulliparous cesarean births (group 2b) that are commonly suggested by medical professionals at the time of delivery. Additionally, sometimes hospitals impose unnecessary CS deliveries on women, claiming they are medically necessary**.** A similar situation has been observed in Brazil’s private sector. Precursors of the high CS rate in the private settings to increase hospital income are the lack of adherence to the national guideline for elective CS, the low use of induction, and abuse of the unconscious interest in CS among new mothers [25]. Eyi et al. reported the same pattern in Turkey. Robson groups 5, 3, 2, 1, 4, and 10, identified as the main contributors to the CS rate, were targeted for interventions. Authors suggested commercialization of the health care system as a public policy directed at the private sector, where CS indication seems not to be driven by medical reasons [26].

Iran’s national health policy for Natural Childbirth Promotion program [3, 17, 18] is to reduce the CS rate through reducing nulliparous cesareans. To achieve this goal, the Iranian Ministry of Health should set its goals on the Robson classification system in auditing the CS rates at the hospital level for Robson groups 1 and 2. This would make an appropriate benchmarking process to compare the obstetrics and perinatal quality of care among maternity units and improve management of normal labour and birth**.** Therefore, developing health literacy among women through counseling in birth preparation classes by midwives with an assessment of the quality of the consultations, empowerment of health care providers, especially obstetricians and residents about physiological birth, and striving toward midwife-led continuity of care can be effective strategies to decrease cesarean births in Iran. In this regard, Blomberg in Sweden introduced a “nine-item list” of structural organizational and cultural changes that led to a persistent reduction in cesarean sections and vacuum extractions among nulliparous women without triggering adverse neonatal outcomes. The nine items include monitoring of obstetric results, recruitment of a midwife coordinator, risk classification of women, the introduction of three different midwife competence levels, improved teamwork, obstetrical morning round, fetal monitoring skills, obstetrical skills training, and public promotion of the strategy [27]. It seems that the Natural Childbirth Promotion program could benefit from a revision based on international best practices.

To our knowledge, this is the first Iranian study to assess CS rates using the Robson classification system based on the national birth registry database. We used a registry database at the national level, as well as a standard classification system, providing a logical comparison with other nations. Another strength of the present study is the comprehensive database of the IMaN that provides information at the hospital, medical university, and national level. This information are vital in implementing strategies and auditing the outcomes at local and national’s levels. This study had several limitations. One limitation of the study is that we did not analyze the maternal and neonatal outcomes in each Robson group. Therefore, the associations between higher CS rates and obstetric outcomes were lacking. Moreover, the Robson criteria on the IMaN registry database were not available before 21 March 2019. It is recommended that future studies focus on maternal and perinatal outcomes and CS rate trends of consecutive years using Robson classification.

## Conclusions

The present study showed that Robson groups 2 and 1 were the second and third largest contributing groups to overall cesarean sections, suggesting the need for further attention and audit. Additionally, there were significant variations in CS rates by hospital peer-group, especially for private maternity units. The results of this study help identify effective strategies to reduce the CS rate in Iran, focusing on the groups that have the main impact on the total CS rate. It also provides appropriate benchmarking to compare obstetric care with other countries with better maternal and perinatal outcomes. The data provided in this study could be useful in a follow-up study to optimize the physiological processes of labour and birth.

## Supplementary Information

**Additional file 1.** The mean CS rates in group 1 for Medical Universities and the number of hospitals affiliated with each university by hospital peer-group.

**Additional file 2.** The mean CS rates in group 2 for Medical Universities and the number of hospitals affiliated with each university by hospital peer-group.

## Data Availability

The datasets used and/or analyzed during the current study are available from the corresponding author on reasonable request.

## Abbreviations

CS: Cesarean Section
WHO: World Health Organization
IMaN: Iranian Maternal and Neonatal
VBAC: Vaginal Birth After Cesarean
