# Supplementary material for: Cesarean delivery in Iran: a population-based analysis using the Robson classification system
Source: BMC Pregnancy Childbirth. 2022 Mar 8;22:185. doi: 10.1186/s12884-022-04517-1 (PMC8903666; doi:10.1186/s12884-022-04517-1)
Supplement: Supplementary file 1 — Additional file 1. The mean CS rates in group 1 for Medical Universities and the number of hospitals affiliated with each university by hospital peer-group. [file 12884_2022_4517_MOESM1_ESM.docx]

**Table S1. The mean CS rates in group 1 for Medical Universities and the number of hospitals affiliated with each university by hospital peer-group ^a^.**

| **Medical University** | **Public** | | **Private** | | **Others** | |
| --- | --- | --- | --- | --- | --- | --- |
|  | **Number of Hospitals** | **CS Rate** | **Number of Hospitals** | **CS Rate** | **Number of Hospitals** | **CS Rate** |
| Abadan | 6 | 24.1 | 0 |  | 2 | 13.2 |
| Ardabil | 10 | 41.7 | 4 | 88.2 | 4 | 17.1 |
| Asadabad | 2 | 30 | 0 |  | 0 |  |
| Azerbaijan Gharbi | 22 | 27.6 | 3 | 66.4 | 4 | 25 |
| Babol | 5 | 31 | 2 | 52.6 | 0 |  |
| Bam | 3 | 15.9 | 1 | 29 | 0 |  |
| Behbahan | 3 | 34.2 | 0 |  | 1 | 36 |
| Birjand | 12 | 23.6 | 0 |  | 4 | 15.1 |
| Boushehr | 8 | 53.3 | 0 |  | 8 | 21.8 |
| Dezfoul | 3 | 24.9 | 0 |  | 3 | 31.9 |
| Esfarayen | 1 | 27.9 | 0 |  | 0 |  |
| Fasa | 1 | 22.7 | 0 |  | 0 |  |
| Gerash | 1 | 28.8 | 0 |  | 0 |  |
| Gilan | 23 | 44.5 | 10 | 38.5 | 1 | 21.8 |
| Golestan | 15 | 24 | 8 | 79.8 | 3 | 22.3 |
| Gonabad | 2 | 20.1 | 0 |  | 0 |  |
| Hamedan | 16 | 33.7 | 1 | 56.8 | 5 | 15.3 |
| Hormozgan | 20 | 18.8 | 3 | 42.97 | 4 | 31.4 |
| Ilam | 10 | 36.2 | 2 | 21.4 | 1 | 33.3 |
| Iran | 17 | 28 | 33 | 54.1 | 10 | 40.2 |
| Iranshahr | 10 | 12.8 | 0 |  | 0 |  |
| Isfahan | 29 | 34.3 | 8 | 48.9 | 9 | 19.7 |
| Jahrom | 2 | 36.2 | 0 |  | 0 |  |
| Jiroft | 6 | 23 | 1 | 11.8 | 0 |  |
| Jondi Shapour Ahvaz | 22 | 33 | 4 | 68 | 14 | 21.6 |
| Karaj | 11 | 40.3 | 5 | 50.8 | 2 | 11.8 |
| Kashan | 7 | 26.3 | 2 | 43.9 | 1 | 28.9 |
| Kerman | 12 | 27 | 2 | 57 | 8 | 31 |
| Kermanshah | 15 | 29 | 2 | 46.3 | 3 | 33.2 |
| Khalkhal | 1 | 36.2 | 0 |  | 0 |  |
| Khomein | 1 | 28.4 | 0 |  | 0 |  |
| Khorasan Shomali | 8 | 23.7 | 0 |  | 2 | 35 |
| Khoy | 3 | 21.5 | 0 |  | 1 | 14.6 |
| Kordestan | 12 | 21.4 | 1 | 40.2 | 3 | 9.3 |
| Larestan | 5 | 24.5 | 0 |  | 0 |  |
| Lorestan | 17 | 35.6 | 5 | 74.3 | 4 | 27.7 |
| Maragheh | 1 | 45.4 | 0 |  | 0 |  |
| Markazi | 13 | 19.2 | 2 | 28.7 | 2 | 24.8 |
| Mashhad | 29 | 18.9 | 6 | 25.5 | 14 | 24.8 |
| Mazandaran | 25 | 36.6 | 7 | 55.4 | 7 | 35.6 |
| Neyshabour | 2 | 8.7 | 0 |  | 1 | 36.1 |
| Qazvin | 10 | 37.9 | 4 | 55.7 | 3 | 16.9 |
| Qom | 9 | 44.5 | 1 | 15.9 | 4 | 26.8 |
| Rafsanjan | 3 | 15.7 | 0 |  | 0 |  |
| Sabzevar | 6 | 9.5 | 0 |  | 0 |  |
| Sarab | 1 | 13.5 | 0 |  | 0 |  |
| Saveh | 3 | 18.8 | 1 | 44.4 | 1 | 19.9 |
| Semnan | 6 | 35.5 | 1 | 55.4 | 2 | 25.8 |
| Shahid Beheshti | 16 | 39.6 | 18 | 37.9 | 23 | 41 |
| Shahid Sadoughi Yazd | 13 | 26 | 4 | 56.1 | 3 | 22.8 |
| Shahrekord | 9 | 37.7 | 1 | 53.5 | 1 | 36.7 |
| Shahroud | 3 | 34.5 | 0 |  | 1 | 60.9 |
| Shiraz | 40 | 41.1 | 14 | 55.4 | 5 | 39.8 |
| Shoushtar | 2 | 28.7 | 0 |  | 1 | 39 |
| Sirjan | 2 | 39.4 | 0 |  | 1 | 46.1 |
| Tabriz | 28 | 32.2 | 7 | 67.5 | 8 | 43.5 |
| Tehran | 9 | 50.8 | 8 | 74.9 | 6 | 38.2 |
| Torbat Heidariyeh | 2 | 22.2 | 0 |  | 1 | 9 |
| Torbat Jam | 2 | 17 | 0 |  | 0 |  |
| Yasouj | 9 | 27.9 | 0 |  | 2 | 8.8 |
| Zabol | 3 | 29.7 | 0 |  | 0 |  |
| Zahedan | 5 | 18.6 | 0 |  | 2 | 9 |
| Zanjan | 10 | 27.6 | 1 | 75.9 | 3 | 18.8 |
| **Total** | **602** | **28.9** | **172** | **50.7** | **188** | **26.8** |

a) Due to the large number of hospitals in this study, the mean CS rates at medical university level by hospital peer-group were reported.
