# Supplementary material for: Cesarean delivery in Iran: a population-based analysis using the Robson classification system
Source: BMC Pregnancy Childbirth. 2022 Mar 8;22:185. doi: 10.1186/s12884-022-04517-1 (PMC8903666; doi:10.1186/s12884-022-04517-1)
Supplement: Supplementary file 2 — Additional file 2. The mean CS rates in group 2 for Medical Universities and the number of hospitals affiliated with each university by hospital peer-group. [file 12884_2022_4517_MOESM2_ESM.docx]

**Table S2. The CS rates in group 2 for Medical Universities and the number of hospitals affiliated with each university by hospital peer-group ^a^.**

| **Medical University** | **Public** | | **Private** | | **Others** | |
| --- | --- | --- | --- | --- | --- | --- |
|  | **Number of Hospitals** | **CS Rate** | **Number of Hospitals** | **CS Rate** | **Number of Hospitals** | **CS Rate** |
| Abadan | 6 | 30.9 | 0 |  | 2 |  |
| Ardabil | 10 | 24.6 | 4 | 98.9 | 4 | 73.8 |
| Asadabad | 2 | 48.2 | 0 |  | 0 |  |
| Azerbaijan Gharbi | 22 | 47 | 3 | 96.3 | 4 | 64.7 |
| Babol | 5 | 57.5 | 2 | 91.1 | 0 |  |
| Bam | 3 | 43.4 | 1 | 63.3 | 0 |  |
| Behbahan | 3 | 42.9 | 0 |  | 1 | 45.2 |
| Birjand | 12 | 40.3 | 0 |  | 4 | 55.3 |
| Boushehr | 8 | 51.8 | 0 |  | 8 | 70.8 |
| Dezfoul | 3 | 37.7 | 0 |  | 3 | 43.6 |
| Esfarayen | 1 | 33.8 | 0 |  | 0 |  |
| Fasa | 1 | 52 | 0 |  | 0 |  |
| Gerash | 1 | 67.4 | 0 |  | 0 |  |
| Gilan | 23 | 60 | 10 | 93.5 | 1 | 45.7 |
| Golestan | 15 | 45.8 | 8 | 93.1 | 3 | 36.9 |
| Gonabad | 2 | 45 | 0 |  | 0 |  |
| Hamedan | 16 | 58 | 1 | 95.5 | 5 | 32.1 |
| Hormozgan | 20 | 41.4 | 3 | 89.2 | 4 | 54 |
| Ilam | 10 | 42.9 | 2 | 49.9 | 1 | 76.1 |
| Iran | 17 | 53.9 | 33 | 93.9 | 10 | 84.6 |
| Iranshahr | 10 | 22.6 | 0 |  | 0 |  |
| Isfahan | 29 | 58.9 | 8 | 88.6 | 9 | 67.3 |
| Jahrom | 2 | 62.8 | 0 |  | 0 |  |
| Jiroft | 6 | 43.5 | 1 | 49.9 | 0 |  |
| Jondi Shapour Ahvaz | 22 | 47.6 | 4 | 95.1 | 14 | 61.7 |
| Karaj | 11 | 45 | 5 | 87.9 | 2 | 38.8 |
| Kashan | 7 | 44.7 | 2 | 80.9 | 1 | 48.3 |
| Kerman | 12 | 60.7 | 2 | 92.8 | 8 | 67.2 |
| Kermanshah | 15 | 57.9 | 2 | 84 | 3 | 35.8 |
| Khalkhal | 1 | 78.3 | 0 |  | 0 |  |
| Khomein | 1 | 49.3 | 0 |  | 0 |  |
| Khorasan Shomali | 8 | 36.2 | 0 |  | 2 | 43.6 |
| Khoy | 3 | 51.9 | 0 |  | 1 | 93.6 |
| Kordestan | 12 | 31 | 1 | 49.8 | 3 | 20.3 |
| Larestan | 5 | 42.3 | 0 |  | 0 |  |
| Lorestan | 17 | 38.7 | 5 | 90.8 | 4 | 36 |
| Maragheh | 1 | 75.3 | 0 |  | 0 |  |
| Markazi | 13 | 33.4 | 2 | 70 | 2 | 34.9 |
| Mashhad | 29 | 37.6 | 6 | 64.7 | 14 | 54.2 |
| Mazandaran | 25 | 58.3 | 7 | 86.4 | 7 | 73.3 |
| Neyshabour | 2 | 54.5 | 0 |  | 1 | 92 |
| Qazvin | 10 | 53.2 | 4 | 80.2 | 3 | 28.2 |
| Qom | 9 | 27.3 | 1 | 84.5 | 4 | 84.9 |
| Rafsanjan | 3 | 68.2 | 0 |  | 0 |  |
| Sabzevar | 6 | 35.7 | 0 |  | 0 |  |
| Sarab | 1 | 33.1 | 0 |  | 0 |  |
| Saveh | 3 | 35.5 | 1 | 80.9 | 1 | 28.2 |
| Semnan | 6 | 57.9 | 1 | 84.3 | 2 | 47.9 |
| Shahid Beheshti | 16 | 78 | 18 | 96.7 | 23 | 90.7 |
| Shahid Sadoughi Yazd | 13 | 45.3 | 4 | 59.4 | 3 | 47.5 |
| Shahrekord | 9 | 49.2 | 1 | 79.3 | 1 | 17 |
| Shahroud | 3 | 63.9 | 0 |  | 1 | 85 |
| Shiraz | 40 | 56.4 | 14 | 84.5 | 5 | 80.8 |
| Shoushtar | 2 | 50.6 | 0 |  | 1 | 30 |
| Sirjan | 2 | 44.5 | 0 |  | 1 | 40.2 |
| Tabriz | 28 | 51.4 | 7 | 93.8 | 8 | 73.4 |
| Tehran | 9 | 49.7 | 8 | 95.7 | 6 | 78.7 |
| Torbat Heidariyeh | 2 | 24.8 | 0 |  | 1 | 21.3 |
| Torbat Jam | 2 | 28.7 | 0 |  | 0 |  |
| Yasouj | 9 | 42.7 | 0 |  | 2 | 23.7 |
| Zabol | 3 | 21.8 | 0 |  | 0 |  |
| Zahedan | 5 | 27.2 | 0 |  | 2 | 57.6 |
| Zanjan | 10 | 41.4 | 1 | 98.2 | 3 | 42.8 |
| **Total** | **602** | **46.7** | **172** | **83.1** | **188** | **53.5** |

a) Due to the large number of hospitals in this study, the mean CS rates at medical university level by hospital peer-group were reported.
